# Social relationships and relational pain in brain tumor patients and their partners

**DOI:** 10.3389/fpain.2022.979758

**Published:** 2022-10-05

**Authors:** Maria L. Boccia

**Affiliations:** Department of Human Sciences and Design, Baylor University, Waco, TX, United States

**Keywords:** brain tumor, social pain, emotional pain, relationships, psychosocial pain

## Abstract

Partners play an important role in both the general well-being and the care needs of patients. The dynamic between brain tumor treatment and patients’ families is a complex bidirectional relationship. Cancer diagnosis and treatments which leave patients compromised impact the nature and quality of their relationships, and these in turn impact the ability of their partners to care for them. This paper will review the nature of the impact of diagnosis and treatment on relationships and how couples and families respond to the disruption of cancer treatments. The impact of how emotional and social pain effect their relationships and their ability to engage in care will be addressed.

## Introduction

When a patient receives a cancer diagnosis, the repercussions extend far beyond the individual. Individuals are embedded in relationships with others and these relationships influence their thoughts, feelings, and behaviors. Families are systems, and the whole system is greater than the sum of the parts. Systems are unique and cannot be understood by looking only at the individuals. Therefore, for the patient, as part of a family system, the diagnosis impacts not only the patient and other members of the family, but also the family system as a whole. Thus, the experience of cancer-related pain, and the management thereof is impacted by how the family system responds, the resources, and coping mechanisms available to not just the patient but to the family. Furthermore, the position of the person in the family as well as vulnerabilities in other members of the family system will impact the patient.

Although cancer research has focused on genetic, immune, and other molecular processes, there has been significant advancement in understanding how the nervous system affects cancer development and recovery (1). The response of the nervous system to stress includes suppression of immune functions such as natural killer cell activity and T-cell proliferation, which play an important role in the immune response to cancer. In addition, stress has been found to inhibit the repair of damaged DNA, which can be a significant component of cancer onset (2). Therefore, understanding the psychosocial effects on the nervous system is growing in importance for cancer care and survivorship. While other types of cancers (such as breast, colon, and prostate) have received attention with regard to psychosocial factors, neuro-oncology patients are under-represented in this research (3, 4).

When referring to pain, it is most common to associate this with physical pain (5–7). Physical pain is associated with unpleasant experiences that are capable of actual or potential physical damage to the individual. Pain captures one’s attention because of its aversiveness, and thereby allows the individual to be aware of the threat to one’s well-being. A great deal is known about the neurobiology of pain due to the discovery and mapping of pain pathways in the nervous system. Emotional pain is also an aversive experience that is less clearly delineated in the scientific literature. However, emotional pain shares the common elements of aversiveness and threat to one’s well-being (5, 8).

Social pain, considered a subtype of emotional pain, is experienced when there is a perceived threat or loss of social relationships (9). Social pain is not just about isolation or being alone. Rather, it is the gap between current social relationships and perceived need of social relationships. Thus, this includes the experience of low social support, social isolation, poor social functioning, and loneliness. In the context of a significant threat, such as a cancer diagnosis, patients will experience a heightened need for social connection as an important component of coping. Lack of or inadequacy of such connections are experienced as social pain and have significant consequences on morbidity and mortality (10).

There is evidence that the neurobiology underlying social and physical pain share common pathways (9). Social threat engages overlapping neural mechanisms with fear and stress responses, including the amygdala as a core structure. Imaging studies have shown that the dorsal anterior cingulate cortex and anterior insula are activated during both physical pain and social pain, including social pain in the context of bereavement, social rejection, and loss of an unborn child (11). Individual differences in sensitivity to social pain have been linked to a gene found to be involved in sensitivity to physical pain, the mu-opioid receptor gene (OPRM1) (12). Pharmacological interventions that are typically thought of as relieving physical pain, such as opioids, are also effective in alleviating social pain (11). There is also some indication that the pharmacological interventions may be differentially effective in relieving social pain in males and females (13).

The interplay between physical, psychological, and social factors in human health and disease has been explored over several decades in the field of psychoneuroimmunology (PNI). While research in this field has consistently found that psychosocial interventions impact stress hormone and immune system function, less attention has been paid to disease progression (14). Given the role of the immune system in cancer and its treatment, one may expect that psychosocial interventions would impact occurrence and progression.

Whether physical or emotional, pain is a stressor, and quality of life (QoL), depression and fatigue are interrelated with pain. There is sufficient evidence to conclude that social support, such as the relationships between patients and their families, can mitigate these stressors (15). In this article, the elements that contribute to the family dynamics and the impact of this on the relationships with the patient will be explored. Where available, this discussion will include studies of brain tumor patients and their partners. There is a gap in our understanding, however, as much of the research in this area has focused on breast, prostate, and other cancers of the reproductive system.

## Impact of cancer diagnosis on relationships

### Patients

Patients are embedded in a system of relationships, and when a diagnosis is received, it is received by the entire system. The patient’s response to that diagnosis, in terms of distress, hope, decisions about treatment, and other aspects of life, is dependent on the entire system’s reaction to it. Partners and family members all respond to the diagnosis and participate in treatment decisions. How families respond to traumas, of which cancer diagnosis is an example, depends on their available resources for coping and whether they perceive their ability to manage the trauma with those resources. Surgical resection itself has been found to be only weakly correlated with changes in QoL for patients (16). A negative perception of their ability to cope can result in significant increases in individual and family stress as well as a breakdown in the family’s ability to maintain relationships and adapt appropriately to stressors and to continue with the necessary tasks of daily life. Psychosocial pain in both patients and their families can be the result, and that pain can both accelerate growth of cancer and impede treatment effectiveness (17–19).

A significant source of anxiety and depression is the fear of cancer recurrence, and this fear impacts both the patient and their partner (20). Muldbücker and colleagues found that, for multiple types of cancer, both patients and partners exhibited fear of recurrence. In brain tumor patients, fear of recurrence is also prevalent, and the caregivers of the patients have reported higher levels of fear of recurrence than the patients themselves (3). High fear of recurrence of the cancer is associated with poorer quality of life, higher levels of distress, and greater functional impairment. Psychosocial and physical pain tend to covary, and patients having elevated pain in both domains also tend to have higher mortality rates (21). There is evidence that psychosocial pain is perceived more negatively than physical pain (22). Thus, attending to psychosocial pain may contribute to patient survival.

### Spouses/partners

Families, particularly spouses, are most often in the role of caregivers for cancer patients. Patients rely heavily on these caregivers for support. Contact with a loved one has been shown to reliably reduce the experience of physical pain and has also been shown to enhance experiencing comfort from their partner during emotionally painful conversations, and to support subsequent adaptive processing of emotional pain (23). Relationship satisfaction in this study moderated the impact of contact on the experience of comfort (23), so the quality of relationships may be a significant factor in the impact of caregiver support.

Brain tumor patients are particularly reliant on social resources because of the impact of tumors and their treatment on brain function and the resultant potential changes in cognition, language, personality and/or emotionality. Furthermore, these changes increase risk of compromised interpersonal relationships which can negatively impact the care the patient receives. Sexual relationships, in particular, impact patients’ QoL, in that the presence of sexual dysfunctions correlates with lower QoL measures in multiple dimensions (24, 25). Sexuality is a vital factor in romantic attachments, and thus in maintaining trust and a sense of security in healthy partner relationships (26, 27). It is not surprising, then, that this represents a significant concern for QoL in patients and their families (24, 28, 29).

The ability of partners who serve as caregivers to sustain healthy communication can impact the burden of care. In situations of long-term care needs, the caregiver burden decreases over time if there is good communication (30). Critical to the burden experience is the concordance in the communication between the patient and caregiver regarding the cancer (31). This does not mean identical perceptions or lack of disagreements, but rather that they are able to communicate openly and effectively. Those who had the highest concordance in communication also reported the highest family cohesion and the lowest caregiver burden. Cohesion, or social connectedness, can contribute to the well-being of caregivers, resulting in less depression in the context of caregiver burden (32). This may reduce the negative impact on caregivers’ ability to provide quality care and thus improve outcomes.

The quality of communication between healthcare providers and patients and their families may be influential in their adaptation to diagnosis, treatment, and even loss. Given the high mortality associated with many forms of brain tumors, it is notable that after the death of the patient, a positive relationship with healthcare providers prior to death leaves caregivers with a positive perception of the last therapies employed (33). This suggests that compassionate communication between providers and families can ease the psychosocial pain associated with the diagnosis and treatment of these cancers, even in the face of the loss of their loved one. Long-term survivors tend to express frustration at functional limitations that do not improve (34), and this adds to the challenges of family caregivers who are concerned about not only care, but adaptation to diminished functioning and patient safety. Depression is associated with a risk of suicidal ideation (35, 36), a feature common in partners both before and after the death of the patient (37). Thus, quality of healthcare providers communication with patients and their families may serve multiple functions in their adaptation to the experience.

### Family members

Friends and families provide significant sources of support that substantially affect patients’ adjustment to their diagnosis (38). Caregivers tend to be first-degree relatives, such as spouse, parent, or child. The type of relationship impacts psychosocial effects on the caregiver: a parent caring for child with cancer experiences very different emotions from an adult child caring for a parent with cancer or a spouse dealing with their partner’s cancer (39–41). Expectations for each of these relationships in terms of intimacy, emotional and other forms of support are quite different, which may result in very different reactions to being in the caregiver role. Because of the distress experienced by both patients and their partners in response to the cancer diagnosis, support from friends may be particularly helpful in the adjustment to the diagnosis and easing psychosocial pain.

Social support for families dealing with cancer diagnosis and treatment significantly affects their ability to cope. Social support includes multiple types of support, including emotional, spiritual, physical, and/or financial. Feeling socially connected can impact the mental health of caregivers, particularly for levels of depression (32). The typical levels of caregiver burdens in terms of time spent providing care (over 30 h/week on average) and financial changes (42) combine to place caregivers in a vulnerable psychosocial position. We know from animal studies that isolation stress is particularly important to address, in that the stress hormones released contribute to the immune dysregulation that contributes to the growth of cancer (43). Although the need for support from friends outside the immediate family may be particularly important, these burdens may interfere with the connections necessary for that support. Therefore, addressing the quality of social support and relationship health may be a potentially important facet of survivorship.

There are several characteristics of families that help us understand whether and how well they will cope with a trauma such as a cancer diagnosis. Olsen’s (44) Circumplex Model integrates these characteristics in such a way as to be useful for understanding family function and ability to manage change, stress, and trauma. Three factors are involved in this model: cohesion, flexibility, and communication. This model assumes that for families, there is no one “right way” to be family. Rather there are a range of family systems which can establish healthy functioning. Cohesion is the emotional connection of the couple and family members with each other. Flexibility is the ability to change in the face of challenges, and includes the ability to alter leadership, roles, rules, negotiation styles, and so on. These two factors can be placed in an orthogonal space, where family systems functioning in the mid ranges of both dimensions are referred to as balanced and are able to maintain healthy functioning. Families who fall outside this midrange (for example, by being very rigid or incoherently flexible) are described as unbalanced and are the families that typically are unable to handle stressors and traumas in healthy ways, often with the result of dysfunction or dissolution. Communication, the third dimension is a facilitating dimension. This means that families are able to communicate effectively to alter their cohesion and flexibility in response to demands placed on the family. Since this model was first proposed, a great deal of research has documented its usefulness in understanding families in a wide variety of circumstances, ranging from unemployment to substance use disorders, to traumatic losses (45–47). How families respond to a patient's cancer diagnosis, then, may be understood if considered in the light of this model. A family in the balanced range may be much more likely to be able to function and support the patient, resulting in better outcomes and QoL, than a family in the unbalanced range.

## Health effects

### Mental health effects

Some aspects of mental health, such as depression, anxiety, anger and hopelessness, have been recognized in patient and survivorship care [e.g., (48–51)]. Psychosocial distress has been identified as a significant factor that impacts treatment decision-making and other aspects of cancer care (52). Other aspects of mental life are less well understood.

Personality has been explored as a way to understand the heterogeneity of response to stress and trauma, particularly in PTSD (53). It plays an important role in risk for infectious diseases and certain forms of cancer (54) but is an understudied area in the management and care of other types of patients. While various personality factors, such as Type C, passivity, and conscientiousness have all been implicated, the common factor appears to be the tendency to suppress negative emotions and avoid conflicts. Alexithymia, the inability to identify and describe feelings, has been found to be related to illness, and cancer diagnosis in particular (54). While patients may adopt an alexithymic strategy to cope with the diagnosis and the painful feelings associated with it, it may also contribute to the onset of disease when it is a stable personality factor. While these intrapersonal traits have been linked to severity of illness, interpersonal personality traits have been linked to treatment effectiveness in PTSD (53). This suggests that attention to both types of individual differences may enhance our understanding of responses to cancer diagnosis as well as to treatment.

### Relational health effects

Traumatic events within the family, such as abuse, family illness or injury, life-threatening accidents, loss of a loved one, or substance abuse, can increase risk of onset or accelerate disease progression. Cancer diagnosis itself is often also experienced as a trauma. As noted above, family functioning impacts their ability to respond to such a diagnosis effectively. The reciprocal, however, also occurs: Experience of a traumatic event may impact the health of the relationships among family members of the patient [e.g., (46)]. Even typical family developmental changes such as the birth of a child can impact the family’s balance (44), shifting flexibility, cohesion, or both. Families who are functioning well will stay within the balanced domain, however, a severe enough challenge can shift the family into an unbalanced position which then may result in family relational dysfunction or dissolution (55, 56). This disruption, then, may impact patient response to treatment as well as survivorship QoL.

Important questions relate to what contributes to both the family system position in the circumplex model as well as what factors lead to families holding these positions. Adverse Childhood Experiences (ACEs) have been identified as powerful predictors of both physical and psychosocial difficulties in adulthood. The original ACEs study (57) was followed by an explosion of research identifying links to adult and family dysfunction and physical illness [see (58) for a review) including mental health diagnoses and increases in morbidity and mortality from all causes. The impact of ACEs on health appears to be dose-dependent: the greater the number of ACEs, the higher the probability of adult problems. It has been estimated that children with the greatest number of ACEs have life expectancies reduced by 20 years (59). This has been so broadly recognized that ACE-screening is routinely used in a variety of contexts such as child protective services, foster care services, adoption agencies, community mental health programs, and hospitals [e.g., (60–63)].

Understanding how ACEs impact adult family function as well as individual morbidity and mortality is crucial to predicting how patients will fare during treatment and disease progression, as well as how the family will respond and be able to support the patients. Animal models have explored ACEs at physiological, genetic, and organismic levels (64). Results indicate that immune dysregulation consequent to the experience of ACEs can impact cancer development and treatment responses. These early experiences may be particularly relevant for brain cancers, in that they have been shown to impact immune function in the central nervous system, including specific areas of the brain such as the hippocampus and midbrain. Thus, a direct link between ACEs and patients and their families’ responses to diagnosis, treatment, and prognosis may be found in the physiological dysregulations experienced by individuals with ACEs in their backgrounds (65–67).

## Interventions

Screening tools for distress in cancer patients have been developed for use across different types of cancers such as lung, breast, and colon cancer (52, 68, 69). Regular screening with referral has been shown to benefit patient treatment as well as survivorship quality of life. Without referrals, however, screening is of little benefit. Specific studies of psychosocial interventions in brain tumor patients, however, are limited. Screening for depression and other mood disorders is complicated by the fact that several criteria, such as fatigue, and appetite and weight loss, can be associated with the disease itself rather than reflection of a mood disorder. Depression must further be distinguished from sadness or grief about declining health. Hospital Anxiety and Depression Scale has been widely used (70, 71). Tools such as The Distress Thermometer (72) or Patient Health Questionnaire (73) have been found to be simple, easy to use, and helpful in identifying patients who need further assessment and/or treatment interventions.

Pain, whether physical or psychosocial, can interfere with healing and recovery of function as well as QoL. Disease progression, treatment decisions and responses, and QoL are all impacted by psychosocial as well as physical pain. Therefore, attention to psychosocial pain must be a component of comprehensive care for cancer patients and their families. The result of treatment is not only associated with decreased distress and improved quality of life, but also with facilitated recovery of function.

Initial responses to a cancer diagnosis are, and must be, addressing decisions about treatment of the cancer itself. However, given how the psychosocial components of a patient’s life impact all of these, addressing these in the comprehensive care plan can improve outcomes. Non-pharmacological cancer treatment may address psychosocial pain and thereby enhance cancer treatment and QoL in survivors, and may include psychotherapy, couple therapy, mindfulness, stress management, and biofeedback (1).

## Conclusions

A cancer diagnosis and subsequent treatment impacts not only the patient, but the entire family system in which the patient is embedded. Brain tumors may be particularly impactful because of the organ system effected. The tumor itself, or treatments such as resection, may impair cognitive, emotional, and other capacities involved in the maintenance and functioning of relationships. The family system responds to this challenge with what perceptions, resources, and coping strategies are available to them. Psychosocial pain is a component of the response when those perceived resources and strategies are insufficient to meet the challenge. Psychosocial pain may be more difficult to identify, measure, and treat than physical pain. However, given the evidence of effects of such pain on disease progression, attending to it may be essential for better outcomes and QoL for patients and their families. Comprehensive cancer care entails a multidisciplinary team of professionals. Assessment of distress is a recommended component of care, but evidence indicates that without referral, just assessing distress is not beneficial (52). Furthermore, consideration of the spouse/partner and family of the patient can significantly affect the process, outcome, and survivorship QoL. It may be helpful to families to be informed about the potential impacts of brain tumors on the patients’ mental capacities. Therefore, inclusion of family members throughout the process by not only including them in discussions but also assessing their distress and relationship with the patient can accomplish several results, including treatment decision-making, psychosocial consequences for the patient and their family, as well as their mental health.

This pain not only impacts quality of life, but also the decisions made in response to the diagnosis, and the effectiveness of treatment. Thus, comprehensive cancer care which includes attention to the family system and to psychosocial pain is likely to have benefits to the patients and their families, both in terms of disease progression and in terms of quality of life in survivorship.
